# A new species of *Characidium* (Characiformes: Crenuchidae) from the Iguaçu National Park, Rio Iguaçu basin, Paraná, Brazil

**DOI:** 10.1111/jfb.70035

**Published:** 2025-04-09

**Authors:** Bruno H. M. Stabile, Renan B. dos Reis, Alessandra V. de Oliveira, Weferson J. da Graça

**Affiliations:** ^1^ Programa de Pós‐Graduação em Ecologia de Ambientes Aquáticos Continentais, Departamento de Biologia Centro de Ciências Biológicas, Universidade Estadual de Maringá Paraná Brazil; ^2^ Núcleo de Pesquisas em Limnologia, Ictiologia e Aquicultura Centro de Ciências Biológicas, Universidade Estadual de Maringá Paraná Brazil; ^3^ Departamento de Biotecnologia, Genética e Biologia Celular, Centro de Ciências Biológicas Universidade Estadual de Maringá Paraná Brazil; ^4^ Programa de Pós‐Graduação em Biologia Comparada, Centro de Ciências Biológicas Universidade Estadual de Maringá Paraná Brazil

**Keywords:** *COI*, conservation unit, freshwater fish, integrative taxonomy, south American darters

## Abstract

A new species of *Characidium* is described from the Iguaçu National Park, Brazil. The new species can be diagnosed from the congeners by the absence of conspicuous vertical bars, blotches and spots along the sides of the body, the presence of a scaled isthmus and adipose fin, a series of scales below lateral line and the presence of a thin dark midlateral stripe. The reticulated colour pattern of melanophores along the posterior edge of scales found in the new species is similar to that found in *C. xanthopterum*, with which it presents 4.2% of genetic distance. Morphological and molecular analyses showed that it is a new species, supported by multiple species delimitation methods (Assemble Species by Automatic Partitioning, the General Mixed Yule Coalescent method, and the Poisson Tree Process and its Bayesian implementation). The new species is a sister group of *C. itarare*, a species that occurs in the Paranapanema river basin (upper Paraná river). Despite being located within a conservation unit, the new species is known from only two creeks, raising concerns about its long‐term conservation.

## 1 INTRODUCTION

Crenuchidae is a family of small freshwater fish, with *Characidium* as its most diverse genus, comprising 86 valid species (Fricke et al., 2025). These fish are primarily distributed in headwater streams across Neotropical basins, extending from Argentina to Panama (Buckup, 2004; Toledo‐Piza et al., 2025).

Among these Neotropical regions, the Rio Iguaçu basin stands out for its remarkable rate of endemism. It harbours 64 endemic species out of a total of 95 (67.4%), making it a biodiversity hotspot. Notably, this basin alone contains more than half of the endemic fish species found in the state of Paraná, Brazil (Baumgartner et al., 2012; Garavello & Sampaio, 2010; Larentis et al., 2016; Mezzaroba et al., 2021; Reis et al., 2020). Nevertheless, despite the considerable diversity observed in the region, only two species of *Characidium* have been documented within this basin, *Characidium travassosi* Melo, Buckup & Oyakawa 2016 and *Characidium* sp. 1 (= *Characidium* aff. *zebra* Eigenmann, 1909 in Ingenito et al., 2004) (Baumgartner et al., 2012; Melo et al., 2016; Mezzaroba et al., 2021).

This basin, recognized as a distinct ecoregion due to the significant divergence in the local fish assemblage and abiotic conditions (Abell et al., 2008), has faced substantial anthropogenic impacts. In recent decades, the construction of a cascade of reservoirs across all segments of the basin, particularly in its lower portion (sensu Ingenito et al., 2004), has profoundly altered its ecosystems (Baumgartner et al., 2012; Larentis et al., 2016; Mezzaroba et al., 2021).

Approximately 18 km above the mouth of the Rio Iguaçu resides one of the highest waterfalls on Earth, the Iguaçu Falls (Stevaux & Latrubesse, 2009). Located on the borders of Argentina and Brazil, the falls have been protected by the Iguaçu National Park since 1939 in Brazil (Brasil, 1939). This park has served as a refuge for terrestrial and aquatic species, protecting several tributaries of the lower Rio Iguaçu from anthropogenic activities (Pini et al., 2021). In the course of recent fieldwork, a new species of *Characidium* was collected in creeks inside the Iguaçu National Park in Brazil. This species is herein described based on both morphological and molecular data.

## 2 MATERIALS AND METHODS

### 2.1 Sample collection

Specimens were collected using a seine net, with sampling authorized by SISBIO (ICMBio), under 14,028–1 license to W.J.G. and the samples were conducted following the policies of the Ethical Conduct Committee on Animal Use (CEUA #7283090823) by the Universidade Estadual de Maringá, Brazil. The collected specimens were euthanized using benzocaine, as per Resolution 1000/2012 of the Federal Council of Veterinary Medicine, Brazil. The fishes were later fixed and preserved in 99% ethanol for molecular studies or set in 10% formalin for morphological studies. The map generated by the sample collection of the specimens of the new species was built on QGIS Development Team (2025).

### 2.2 Morphological data

Morphometric data were taken point to point with a digital calliper (precision of 0.1 mm). The measures are expressed as percentages of standard length (*L*s), except for subunits of the head, which are given as percentages of head length (*L*_H_). Morphometric and meristics data were taken on the left side of the specimens following Buckup (1993), and Melo and Oyakawa (2015). Buckup (1993) defines the count of scales below lateral line being the “number of series of scales between lateral series and midventral line”. To ensure consistency with other species, a similar count was conducted for *C. xanthopterum* (paratype NUP 4414). Counts could not be carried out on some specimens due to conservation limitations (loss of scales). For osteological data, two paratypes were cleared and stained (c&s) according to the procedures of Taylor and Van Dyke (1985). Nomenclature of the osteological structures follows Weitzman (1962). In the description section, each meristic character is followed by the number of specimens examined in parentheses, and the counts of the holotype are identified with an asterisk.

### 2.3 Molecular data

Muscle tissue from specimens previously fixed in ethanol (paratypes used in molecular analyses are identified with “mol.”) were used for DNA extraction using the Wizard Genomic DNA Purification kit (Promega), following the manufacturer's protocol and subsequently quantified with NanoDrop™ Lite Spectrophotometer.

The amplification of a partial fragment of the cytochrome *c* oxidase subunit I (*COI*) through polymerase chain reaction (PCR) was made using primers FishF1 5′‐TCAACCAACCACAAAGACATTGGCAC‐3′ (Ward et al., 2005) and FR1d 5′‐CACCTCAGGGTGTCCGAARAAYCARAA‐3′ (Ivanova et al., 2007). The PCR reaction was performed in a mix containing Tris‐KCl 1× reaction buffer (20 mM Tris–HCl [pH 8.4], 50 mM KCl), MgCl_2_ (1.5 mM), primers (2.5 μM each), dNTPs (0.1 mM each), 1 U Taq DNA polymerase, 10 ng of template DNA and ultrapure water in a final volume of 25 μL. The PCR conditions were as follows: initial denaturation at 95°C for 5 min, followed by 35 cycles at 94°C for 30 s, 52°C for 40 s and 72°C for 1 min, with a final extension at 72°C for 10 min (adapted from Ivanova et al., 2007). The resulting amplicons were later purified using polyethylene glycol 8000 (Rosenthal et al., 1993) and sequenced using an AB™ Sanger Sequencing 3500 Genetic Analyser.

The generated sequences were edited using BioEdit (Hall, 1999) and aligned in MEGA X (Kumar et al., 2018) by ClustalW. Additional sequences from congener species (groups H and I1 according to Oliveira‐Silva et al. (2024)) and *Melanocharacidium blennioides* (Eigenmann, 1909) as an outgroup were incorporated into the final alignment. MEGA X was used to calculate genetic distances (Kimura 2‐Parameter model) and construct a maximum likelihood (ML) tree with 1000 replicates. A final tree was edited using Tree Of Life (iTOL) (Letunic & Bork, 2024).

The two sequences generated in this study for the new species were deposited in GenBank (PQ407555‐PQ407556). Access to the genetic heritage of these species was granted by the National System for Management of Genetic Heritage and Associated Traditional Knowledge (SISGEN, registration A211682). All sequences used in molecular analysis are listed in Table S1.

Furthermore, we generated a Bayesian tree for the General Mixed Yule Coalescent model (GMYC) species delimitation test (see next section) without the outgroup. As one of the premises for the analysis, we used unique haplotypes for the anteriorly chosen sequences, which improved the algorithm and maximized the computational time analysis (Mateussi et al., 2019). The tree was built on an arbitrary timescale and estimated in the BEAST 1.8.4 (Drummond et al., 2012), using an uncorrelated relaxed molecular clock (lognormal) with a birth‐death speciation model. A random tree was used as a starting point for MCMC searches, and two independent runs of 15,000,000 generations with trees sampled at every 1500th generation were estimated. Chain convergences for the two runs were observed at Tracer 1.6 to determine the stationary phase and an effective sample size >200 (Rambaut et al., 2018). 10% of the trees were discarded as a burn‐in procedure in Tree Annotator v. 1.8.4 and the maximum clade credibility tree (MCC) was selected (Drummond et al., 2012). The final tree was observed in the program FigTree v.1.4.4 and generated a newick tree file.

### 2.4 Species delimitation

In addition to the distances calculations and the construction of gene trees, four species delimitations tests were performed to ensure the molecular validity of the new species: Assemble Species by Automatic Partitioning (ASAP) (Puillandre et al., 2021); Poisson tree process (PTP) and its Bayesian implementation (bPTP) (Zhang et al., 2013) and the GMYC method (Fujisawa & Barraclough, 2013).

The ASAP analysis was conducted using the K80 substitution model with default parameters. Both PTP and bPTP analyses utilized the previously constructed ML tree and 300,000 Markov chain Monte Carlo (MCMC) generations, with all other settings kept at their defaults. From the GMYC test, the ultrametric tree in an arbitrary timescale (Newick format, see Molecular data) was used as the input file at the webserver (https://species.h-its.org/gmyc/), using the single threshold parameter.

## 3 RESULTS

### 3.1 *Characidium dumonti*, sp. nov.

urn:lsid:zoobank.org:pub:1CA65975‐F89B‐4E17‐A8BD‐F3A233C1388F.

urn:lsid:zoobank.org:act:F02CFC3A‐EC08‐4DD4‐9555‐FD22D0C7D230.

*Characidium* sp. 1. Larentis et al. (2016): 5 (checklist from streams of the lower Iguaçu River basin; *partim*: CIG 1410). Table 1 in p. 5.

**TABLE 1:** Morphometric data of holotype and paratypes of *Characidium dumonti* (*n* = 20), range includes the holotype.

	Holotype	*n*	Range	Mean	SD
Total length (mm)	61.15	20	40.5–68.9	–	–
Standard length (mm)	49.9	20	31.4–56.4	–	–
Percentage of standard length					
Head length	23.7	20	22.6–26	24.3	1.0
Predorsal distance	45.1	20	44.5–48	46.5	1.1
Prepectoral distance	23.5	20	21.4–26	23.0	1.3
Prepelvic distance	49.8	20	46.6–52.1	49.6	1.4
Preanal distance	77.1	20	72.9–79.8	76.1	1.7
Anal‐apex distance	95.1	20	92–97.8	94.9	1.7
Dorsal‐fin height	21.1	20	19.1–23.7	20.9	1.3
Dorsal‐fin base	15.0	20	12.6–16.3	14.7	0.9
Pectoral‐fin height	21.3	20	19.5–24.6	21.7	1.4
Pelvic‐fin height	13.3	20	12.1–18.1	14.5	1.3
Adipose‐fin height	4.3	20	3.9–6.7	5.1	0.7
Anal‐fin height	15.5	20	13.8–17.8	15.8	1.2
Anal‐fin base	7.8	20	7–9.9	8.0	0.7
Anus to anal‐fin distance	8.0	20	6.3–9	7.6	0.8
Peduncle length	16.3	20	16.3–20.9	18.8	1.2
Body depth at dorsal‐fin origin	23.9	20	19.2–23.9	22.0	1.3
Body depth at anal‐fin origin	16.3	20	13.7–17.5	15.6	1.0
Body depth at caudal peduncle	11.0	20	9.4–11.9	10.7	0.6
Body width	11.4	20	8.3–11.4	9.6	0.7
Percentage of head length					
Snout length	24.3	20	20.1–25.2	22.8	1.6
Snout‐maxillary tip	21.0	20	18–23.9	20.8	1.5
Anterior naris‐orbit	11.1	20	8.2–11.1	9.8	0.8
Posterior naris‐orbit	8.1	20	6.8–11.3	8.3	0.9
Cheek depth	10.5	20	8.5–12.1	9.9	1.1
Orbital diameter	24.2	20	23.6–29	25.9	1.6
Interorbital distance	18.7	20	13.2–19	16.3	1.9

Abbreviation: SD, standard deviation.

**FIGURE 1:**
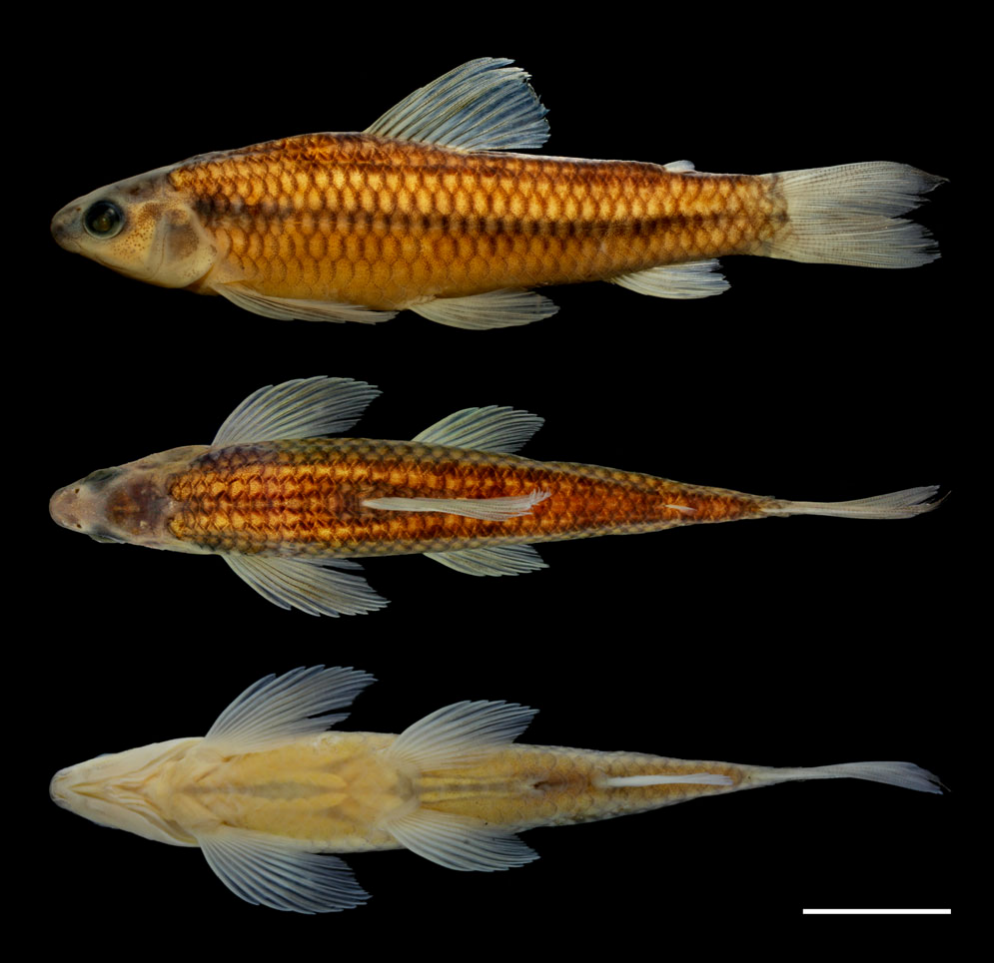
*Characidium dumonti* holotype. NUP 25700, 49.9 mm *L*_S_, Brazil, Paraná, Foz do Iguaçu, Córrego Carlos Giovanni, tributary of Rio São João, Rio Iguaçu basin, Lower Rio Paraná. Lateral view was taken from the right side of the specimen and horizontally mirrored. Scale bar = 10 mm.

**Holotype:** NUP 25700, 49.9 mm *L*_S_, Foz do Iguaçu, Paraná State, Brazil, Córrego Carlos Giovanni, tributary of Rio São João, Rio Iguaçu basin, Lower Rio Paraná, 25°36′47.3″S, 54°25′52.2″W, 16 September 2023, B. H. M. Stabile, R. B. Reis, M. Z. Roloff, C. E. V. Grou, L. D. Lima, S. K. Utiyama & N. P. Lopes (Figure 1).

**Paratype:** All from Brazil, Paraná State, tributary of Rio São João, Rio Iguaçu basin, Lower Rio Paraná, municipality of Foz do Iguaçu:

MCP 55324, 3, 39.3–49.6 mm *L*_S_, Córrego Carlos Giovanni, 25°36′46.4″S, 54°25′55.8″W, 18 April 2010, GERPEL staff. MZUEL 23751, 3, 38.0–51.7 mm *L*_S_, same data as MCP 55324. NUP 18068, 3, 34.8–40.6 mm *L*_S_, unknown name stream, 25°37′20.0″S, 54°26′53.0″W, 25 November 2014, GERPEL staff. NUP 24851, 1, 44.7 mm *L*_S_, mol, same data as holotype. NUP 25044, 1, 43.5 mm *L*_S_, mol, same data as holotype. NUP 25445, 4, 36.7–56.4 mm *L*_S_, same data as MCP 55324. NUP 25446, 4, 31.4–37.6 mm *L*_S_, Córrego Carlos Giovanni, 25°36′44.9″S, 54°25′53.7″W, 30 August 2010, GERPEL staff. NUP 25699, 2 (c&s), 38.4–45.07 mm *L*_S_, same data as NUP 18068.

### 3.2 Diagnosis

*Characidium dumonti* can be distinguished from its congeners, except *Characidium bolivianum* Pearson 1924, *C. chicoi* da Graça, Ota & Domingues 2019, *C. iaquira* Zanata, Ohara, Oyakawa & Dagosta 2020, *C. kamakan* Zanata & Camelier 2015, *C. lanei* Travassos 1967, *C. nana* Mendonça & Netto‐Ferreira 2015, *C. samurai* Zanata & Camelier 2014, *C. summus* Zanata & Ohara 2015, *C. tapuia* Zanata, Ramos & Oliveira‐Silva 2018, and *C*. *xanthopterum* Silveira, Langeani, da Graça, Pavanelli & Buckup 2008 by the absence of conspicuous dark‐brown vertical bars, blotches and spots along the sides of body (vs. presence of conspicuous dark‐brown vertical bars, blotches and spots along the sides of body in the remaining congeners). *Characidium dumonti* can be distinguished from *C. bolivianum*, *C. iaquira*, *C. kamakan*, *C. lanei* and *C. summus* by the scaled isthmus (vs. scaleless isthmus). From *C. chicoi* and *C. nana* by the presence of adipose fin (vs. absence of adipose fin). From *C*. *xanthopterum* by the presence of five scales series below lateral line (vs. four). From *C. samurai* and *C. tapuia* by the presence of a thin dark midlateral stripe occupying half scale height in vertical through dorsal‐fin origin and one scale height on caudal peduncle (vs. broad lateral stripe occupying at least one scale height). The new species can be further distinguished from all congeners except *C. xanthopterum* by the presence of a reticulated colour pattern, with melanophores along the posterior edge of the scales (vs. absence of a reticulated colour pattern, without melanophores along the posterior edge of the scales) and from *C. xanthopterum* by the shorter dorsal and pelvic‐fin height, 19.1%–23.7% and 12.1%–18.1% of *L*s (vs. 24.9%–32.2% and 21.6%–27.4% of *L*s), and shorter predorsal distance 44.5%–48.0% of *L*s (vs. 49.2%–56.7% of *L*s).

### 3.3 Description

Morphometric data of holotype and paratypes in Table 1. Body elongate. Dorsal profile of body straight to slightly convex between anterior tip of snout and end dorsal‐fin base. Straight at end of dorsal fin base to origin of dorsal procurrent caudal‐fin rays. Ventral profile of body convex from lower lip to pelvic‐fin origin, slightly concave to straight between pelvic and anal‐fins origin, and concave between anal‐fin origin to origin of ventral procurrent caudal‐fin rays. Highest body depth on dorsal‐fin origin. Snout triangular‐shaped in lateral view and dorsal view and anterior margin rounded in dorsal view. Mouth subterminal, aligned or lower than ventral margin of orbit. Distal tip of maxilla aligned or almost reaching vertical line through anterior margin of orbit. Orbit approximately circular, larger than snout length. Cheek broad, depth less than a third of orbit diameter. Nares separated, anterior naris approximately equidistant from posterior naris and from orbit. Anterior naris raised, forming circular rim.

Lateral line completely pored, with 34 (4) and 35* (11) scales; horizontal scale rows above lateral line 4* (20); horizontal scale rows below lateral line 5* (20). Scales along middorsal line between supraoccipital and origin of dorsal fin 10* (17) or 11 (3). Scale rows around caudal peduncle 12* (20). Three (3), four (12) or five* (5) scales between anus aperture and anal‐fin insertion. Scaled isthmus.

Dorsal‐fin rays ii, 9* (20); distal margin of dorsal fin straight, anterior and posterior region rounded. Adipose fin well‐developed. Pectoral‐fin rays 11*–12 total rays; iii, 8* (12), iii, 8, i (6) or iii, 9 (2); second and third branched pectoral‐fin rays usually longest; posterior tip of pectoral fin reaching one or two scales anteriorly of pelvic‐fin origin. Pelvic‐fin rays i, 8* (20); second and third branched pelvic‐fin rays longest; posterior tip of pelvic fin surpassing anus. Anal‐fin rays ii, 6* (19) or ii, 7 (1); posterior margin of anal fin straight or rounded, second and third branched ray usually longest. Caudal‐fin rays i, 8, 8, i (1) or i, 9, 8, i* (19).

**FIGURE 2:**
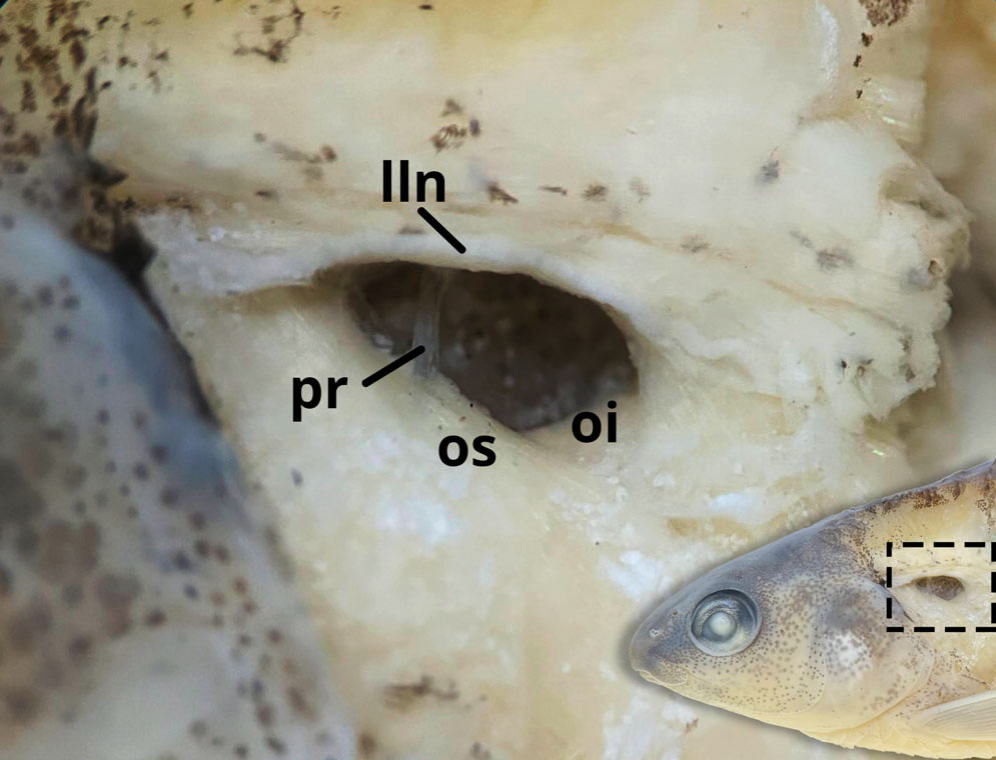
Lateral view of pseudotympanum of *Characidium dumonti* (paratype NUP 24851, 44.7 mm *L*_S_). lln, lateral‐line nerve; oi, *obliquus inferioris*; os, *obliquus superioris*; pr, pleural rib of the fifth vertebrae.

Pseudotympanum present, limited by *lateralis superficialis* in dorsal edges, *obliquus inferioris* in anterior and posterior edges and *obliquus superioris* in ventral edges. Humeral hiatus drop‐shaped, divided into anterior and posterior chambers by pleural rib of fifth vertebra (Figure 2).

**FIGURE 3:**
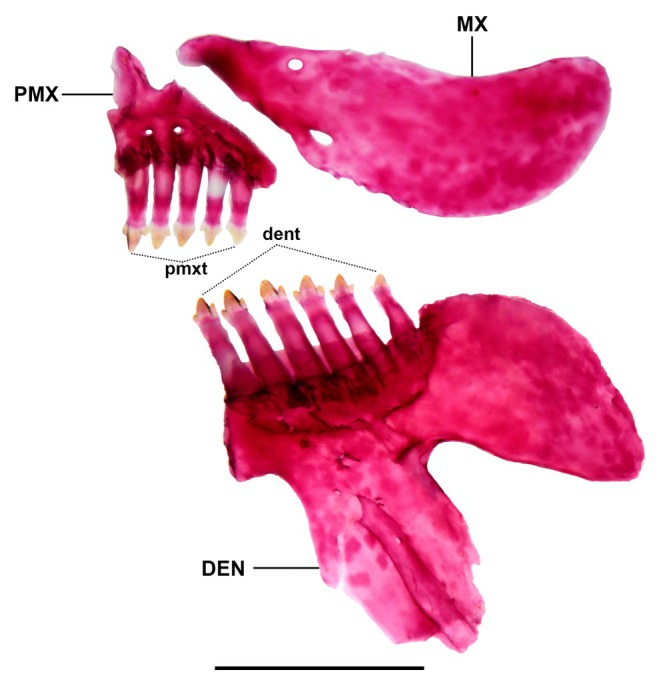
Osteological characteristics of right upper and lower jaw in *Characidium dumonti* (paratype NUP 25699, 45.1 mm *L*_S_). DEN, dentary; dent, dentary teeth; MX, maxilla; PMX, premaxilla; pmxt, premaxillary teeth.

Dentary teeth with single row of 6* (5), 7 (14) or 8 (3) teeth, anterior three or four teeth from symphysis tricuspid, with middle cuspid larger than lateral cuspids; teeth decreasing in size cusps from symphysis. Premaxilla with single row of 6* (18), 7 (3) or 8 (1) teeth decreasing in size from symphysis; larger teeth tricuspid with almost imperceptible lateral cusps. Maxillary toothless (Figure 3). Ectopterygoid with one row, with 6 (1) or 7 (1) minute and conical teeth. Endopterygoid teeth absent. Branchiostegal rays 5 (2), 4 connected to anterior ceratohyal, 1 connected to posterior ceratohyal. Supraorbital triangular in shape, medial margin concave, lateral margin convex, anterior and posterior tips rounded. Parietal branch of supraorbital canal present, extending beyond frontal–parietal border. Parietal fontanel anteriorly limited by frontals. Nasal bones restricted to ossified canal.

Dorsal procurrent caudal‐fin rays 8 (1) or 9 (1); ventral procurrent caudal‐fin rays 6 (1) or 7 (1). Total vertebrae 34 (2); precaudal vertebrae 18 (1) or 19 (1); caudal vertebrae 15 (1) or 16 (1). Supraneural bones 5 (1) or 6 (1). Epural bones 2 (2). Uroneural bones 2 (2). Gill rakers on first arch 11 (2).

### 3.4 Colour in alcohol

**FIGURE 4:**
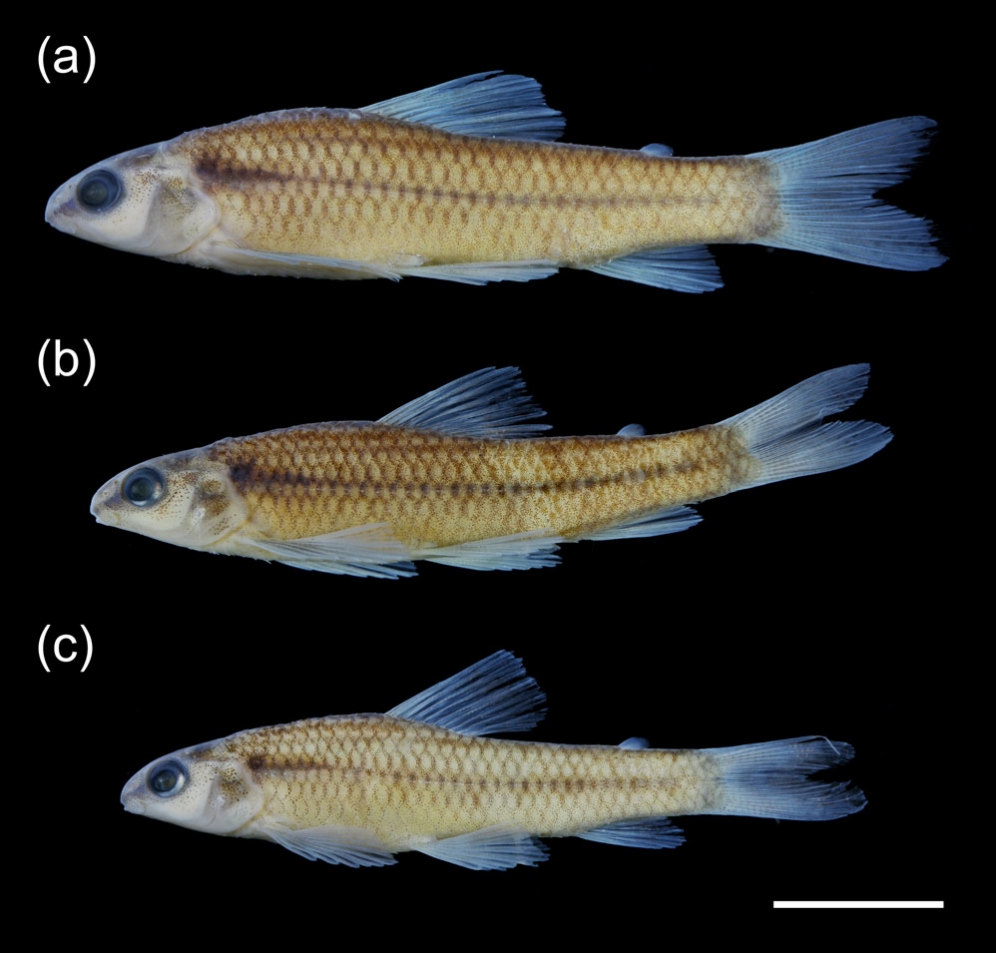
Paratypes of *Characidium dumonti*. (a) NUP 25445, 42.5 mm *L*s; (b) NUP 25446A, 37.6 mm *L*_S_; (c) NUP 25446B, 34.6 mm *L*s. Scale bar = 10 mm.

Background colour of body and head pale yellow. Dark‐brown melanophores in head concentrated at dorsal and clearer in lateral regions, around orbit, opercle and upper lip. Infraorbital and gular regions pale yellow, with few dark‐brown melanophores. Interorbital region with dark‐brown rounded blotches, dorsally in orbit. Dark preorbital stripe, discontinued posteriorly of margin of eye to humeral spot. Rounded to longitudinal elliptical humeral spot, formed by conspicuous dark‐brown melanophores. Dark‐brown, conspicuous and thin (half scale depth) mid‐lateral stripe extending from humeral spot to inconspicuous on last three scales anteriorly to caudal‐fin rays, at level of lateral line and occupying one scale height on caudal peduncle region. Dark‐brown basicaudal spot conspicuous to inconspicuous in some specimens. Two conspicuous dark‐brown longitudinal dorso‐lateral stripes, visible on lateral view, and one conspicuous dorso‐sagittal stripe, visible on dorsal view (all 1/3 scale depth). First dorso‐lateral stripe originated from supraoccipital region and ended on origin of dorsal‐fin. Second dorso‐lateral stripe originated below the first dorso‐lateral stripe and vertically through the middle of pectoral fin, and ended vertically on origin of adipose‐fin. Dorso‐sagittal stripe originated from supraoccipital region and ended on origin of dorsal‐fin. Dark‐brown melanophores along posterior edge of scales, forming reticulate pattern, absent in abdominal region. Bars, blotches or spots absent along the sides of body. Interradial membranes of all fins hyaline, with melanophores slightly concentrated on rays. Adipose fin hyaline, with few melanophores posteriorly (Figure 4).

### 3.5 Colour in life

**FIGURE 5:**
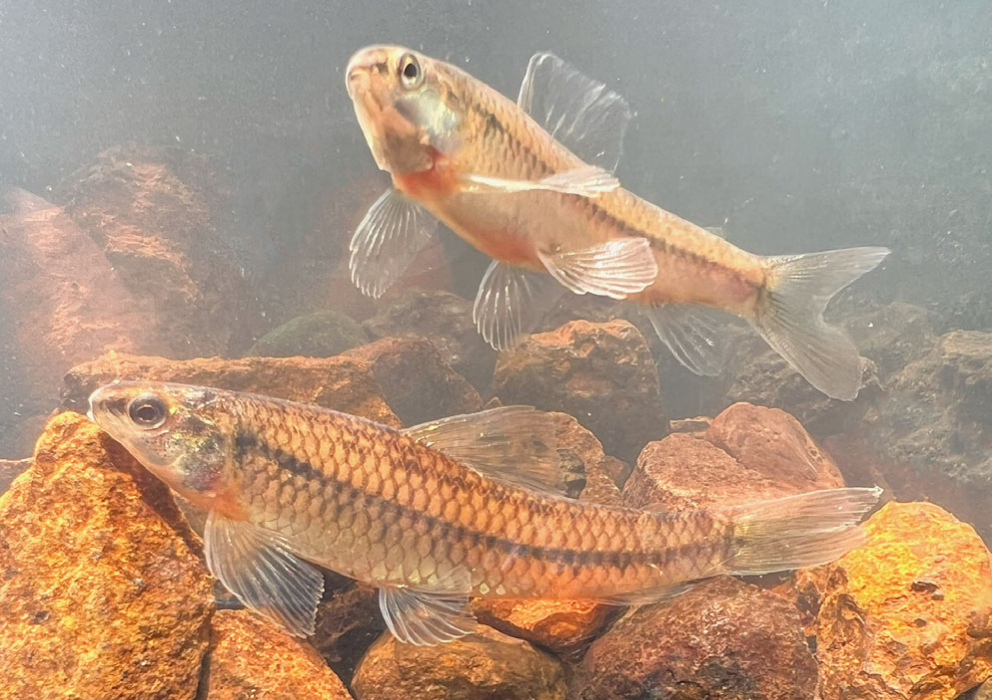
Live specimens of *Characidium dumonti* before fixation, sampled in Córrego Carlos Giovanni, tributary of Rio São João, Rio Iguaçu basin, Lower Rio Paraná. Photograph by Natália de Paula Lopes.

Background colour of body and head yellow (Figure 5). Same pattern as colour in alcohol, except for hyaline fin without apparent melanophores on rays. Iris, preopercle and opercle with golden hues. Conspicuous dark‐brown blotch vertically elongated (four to five scales depth) in base of caudal‐fin rays. Conspicuous basicaudal spot.

### 3.6 Sexual dimorphism

No external sexual characters were observed on the specimens analysed.

### 3.7 Distribution

**FIGURE 6:**
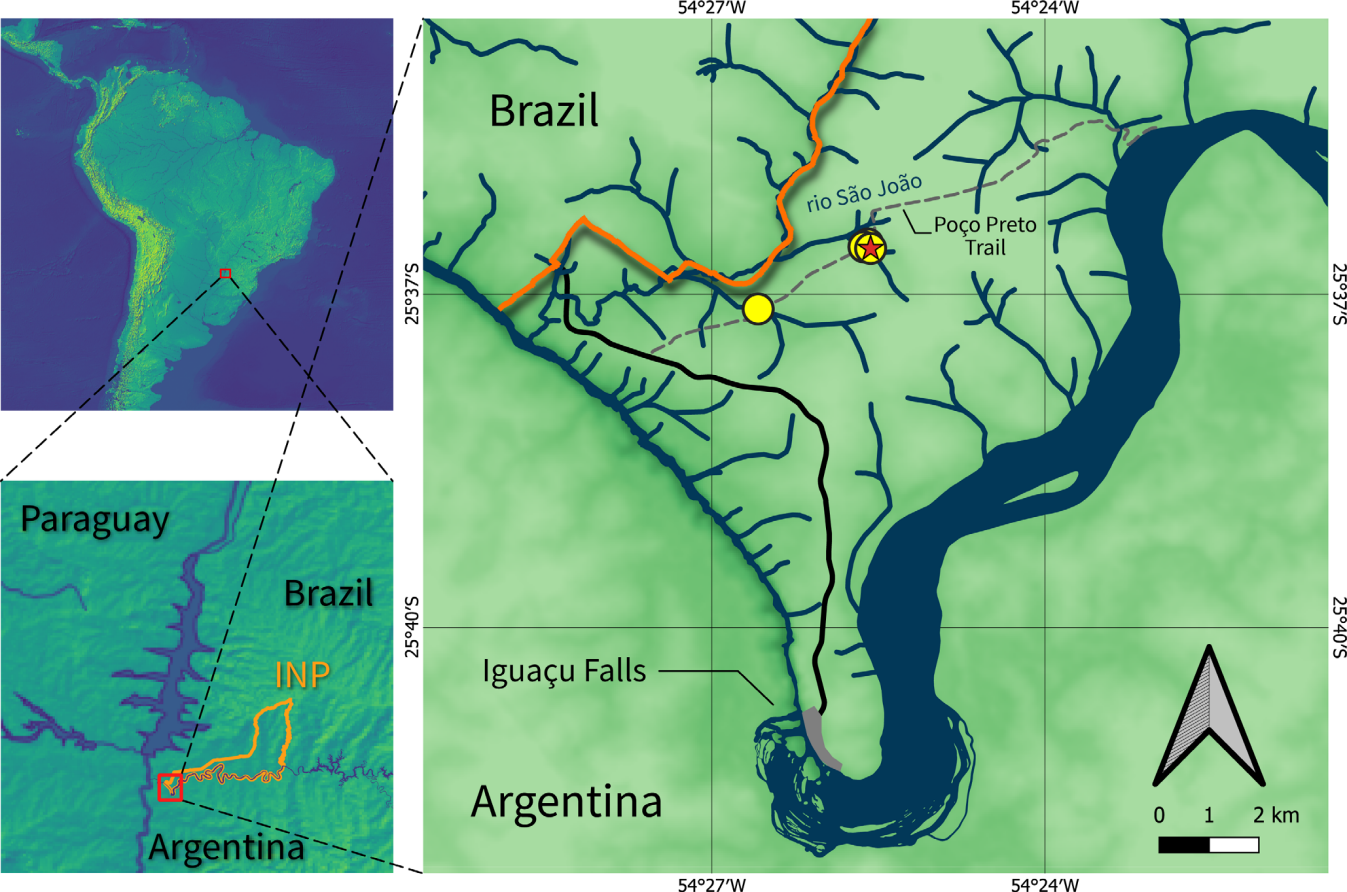
Map of the distribution of *Characidium dumonti*, inside Iguaçu National Park (INP‐orange), Rio Iguaçu, lower Rio Paraná basin, Paraná, Brazil. The yellow circles represent localities of paratypes and the red star represents the type locality.

*Characidium dumonti* is known from two small creeks inside Iguaçu National Park, in tributaries of Rio São João, Rio Iguaçu, lower Rio Paraná basin, Paraná, Brazil (Figure 6).

### 3.8 Ecological notes

**FIGURE 7:**
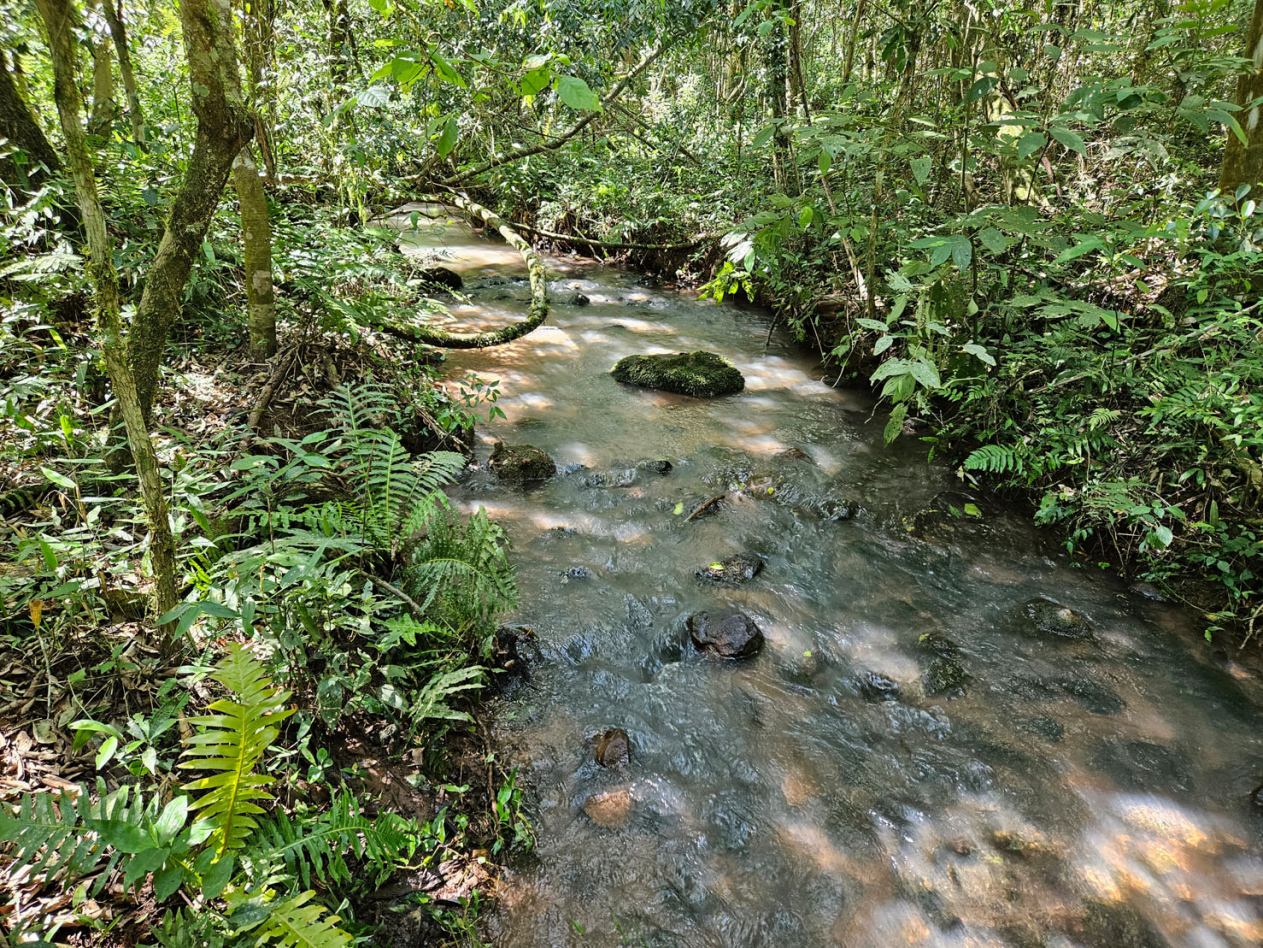
Type locality of *Characidium dumonti*, Córrego Carlos Giovanni, tributary of Rio São João, Rio Iguaçu basin, Lower Rio Paraná system, Paraná, Brazil.

The type locality of *Characidium dumonti* is a fast‐flowing stream in the Iguaçu National Park (Figure 7). The substrate is mainly composed of rocks and pebbles of varying sizes over sandy soil. The vegetation around the streams is a well‐preserved remnant of the Atlantic Forest (Mata Atlântica biome in Brazil), with tracks of large mammals such as tapirs (*Tapirus terrestris*). The following species are sintopic with the new species: *Ancistrus mullerae* Bifi, Pavanelli & Zawadzki 2009, *Cambeva* aff. *stawiarski* (Miranda Ribeiro 1968), *Hoplisoma* cf. *carlae* (Nijssen & Isbrücker 1983), *Hisonotus yasi* (Almirón et al., 2004), *Phalloceros harpagos* Lucinda 2008, *Psalidodon bifasciatus* (Garavello & Sampaio, 2010) and *Psalidodon minor* (Garavello & Sampaio, 2010).

### 3.9 Conservation status

*Characidium dumonti* is endemic to the Rio Iguaçu basin. Despite extensive sampling efforts in tributaries near its type locality within the Iguaçu basin (e.g. Casciotta et al., 2016; Larentis et al., 2016) and adjacent basins, such as the Piquiri (e.g. Cavalli et al., 2018), the new species has only been observed in two sites (Figure 6). These localities, both within the boundaries of the Iguaçu National Park, represent an Area of Occupancy (AOO) of 8 km^2^ (criteria B2, <2000 km^2^). The presence of *C. dumonti* within a federal well‐conserved protected area suggests it currently faces no immediate threats or conditions that would qualify for an IUCN threatened category (according to criteria B2). However, the limited data on population size and distribution, coupled with the restricted sampling locations, hinder a comprehensive assessment of potential risks to the species. In accordance with IUCN guidelines (IUCN Standards and Petitions Committee, 2024), *Characidium dumonti* is hereby suggested to be classified as data deficient (DD).

### 3.10 Etymology

The specific name *dumonti* was chosen in honour of Alberto Santos‐Dumont (1873–1932), who is regarded as the father of aviation. In 1916, Dumont visited the area around the Iguaçu Falls and worked to convince the government to create a natural park there. His efforts were essential for the establishment of the Iguaçu National Park, a place that protects the type locality of *Characidium dumonti* and home of many other species. A genitive.

### 3.11 Molecular analysis

The final alignment comprised 60 sequences and a length of 608 bp, including 179 variable sites of which 154 were parsimony informative. The nearest K2P genetic distances values were 3.0% with *Characidium itarare* Stabile, Reis, Frota, Graça & Oliveira 2024 and 4.1%–4.4% with *Characidium xanthopterum*, both from the Upper Paraná River (Table S2).

**FIGURE 8:**
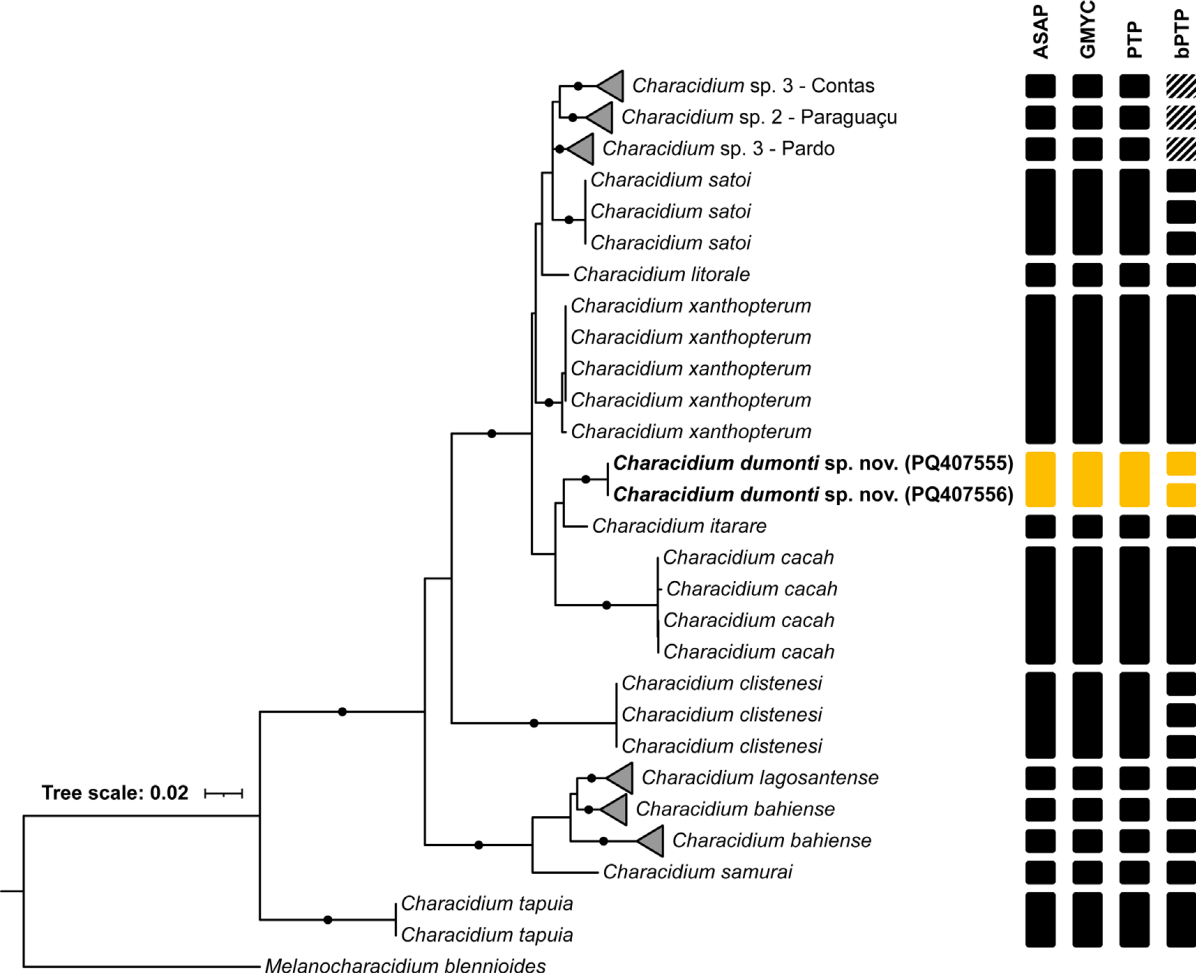
Maximum likelihood gene tree constructed using partial fragment of *COI* sequences with the K2 + G nucleotide substitution model. Circles represent bootstrap values >80. Vertical bars indicate the results of species delimitation methods, Assemble Species by Automatic Partition (ASAP), Generalized Mixed Yule Coalescent (GMYC), Poisson tree process (PTP) and its Bayesian implementation (bPTP). Dashed rectangles indicate multiple OTUs for collapsed clades. *Characidium dumonti* shown in bold. *Melanocharacidium blennioides* was used as an outgroup.

The ML tree topology indicates that *Characidium dumonti* is part of clade H (Oliveira‐Silva et al., 2024). The species is a sister group of *Characidium itarare*, forming a monophyletic clade with *Characidium cacah* Zanata, Ribeiro, Araújo‐Porto, Pessali & Oliveira‐Silva. Of the species delimiters tested, the ASAP, GMYC and PTP methods indicated identical OTUs for all species tested and supported the recognition of *Characidium dumonti* as a new species (Figure 8).

## 4 DISCUSSION

*Characidium dumonti* can be diagnosed from almost all congeners by the absence of bars and blotches on the lateral of the body, and the presence of dark‐brown melanophores along the posterior edge of scales, forming reticulate pattern. The only congener that inhabits the La Plata River basin and exhibit these characters is *C*. *xanthopterum* (from the Upper Paraná and Tocantins‐Araguaia ecoregions, see Silveira et al., 2008 for more details on range distribution), with a genetic distance of 4.1%–4.4%. Furthermore, the other congeners that have the presence of such similar features, *C. kamakan*, *C. tapuia* and *C. samurai*, are geographically (Parnaíba and Northeastern Mata Atlantica ecoregions) and genetically distant (20.1%, 15.6% and 12.6%, respectively) (Zanata et al., 2018; Zanata & Camelier, 2014). Thus, the presence of such characters in distant phylogenetic clades shown in Figure 8 could suggest homoplasy in the colour pattern character.

Molecular analyses revealed a greater genetic similarity (K2P genetic distance value of 3%) between *C. dumonti* and *C. itarare*, a species recently described from the Upper Paraná ecoregion. Additionally, the position of *C. dumonti* within the H group (sensu Oliveira‐Silva et al. (2024)) highlights its phylogenetic proximity to other species in the group, which share the presence of a single row of teeth in the dentary. This finding underscores the need for integrated approaches that combine morphological and genetic data to refine species delimitation and resolve phylogenetic relationships within *Characidium*, especially the biogeographic history of this genus (Agudelo‐Zamora et al., 2020; Oliveira‐Silva et al., 2023; Malanski et al. 2019; Oliveira‐Silva et al., 2024; Stabile et al., 2024).

This species is only the second of the genus to be described from the Rio Iguaçu basin. However, its restricted occurrence in a small stream within a reserve covering approximately 3500 km^2^ along with other adjacent protected areas (Di Bitetti et al., 2006) highlights the lack of knowledge about the local ichthyofauna, particularly when considering the endemicity of this basin (Reis et al., 2020). Despite the challenges faced in recent years, which put its integrity at risk (Prasniewski et al., 2024), the Iguaçu National Park has been playing a crucial role in the conservation of species with restricted distributions that are threatened (Xavier da Silva et al., 2018).

## 5 COMPARATIVE MATERIAL

### 5.1 Characidium heirmostigmata

Upper rio Paraná basin: NUP 1393 (Paratype), 45.3–50.0 mm *L*_S_, municipality of Jussara, Paraná, Brazil, rio Abelha, tributary of rio Ivaí, 23°36′S 52°28′W, Nupélia staff, 17 November 1994.

### 5.2 Characidium itarare

Upper rio Paraná basin: NUP 1393 (Holotype), 24901, 47.9 mm *L*_S_, municipality of Jaguariaíva, Paraná, Brazil, unknown name stream, rio Itararé basin, 24°29′34″S, 49°29′54″W, Graça, W. J. *et al*., 19 July 2018.

### 5.3 Characidium kamakan

Rio Pardo basin: MZUSP 115000 (Holotype), 51.9 mm *L*s, municipality of Camacan, Bahia, Brazil, rio Panelão on the road between Camacan and Jacareci, tributary of rio Pardo, 15°25′16″S 39°31′48″W, Zanata, A. M. *et al*., 15 September 2013.

### 5.4 Characidium onca

Upper rio Paraná basin: MZUSP 125807 (Holotype), 40.1 mm *L*_S_, municipality of Brasília, Distrito Federal, Brazil, córrego Taquara at Reserva Ecológica do IBGE, tributary of ribeirão Gama, tributary of rio São Bartolomeu, 15°54′55.04″S 47°54′23.87″W, Melo, M. R. S. & Ribeiro, M. C. L. B., 14 November 2016.

### 5.5 Characidium pterostictum

Ribeira de Iguape basin: NUP 6712, 3, 38.3–50.0 mm *L*_S_, municipality of São Miguel Arcanjo, Ribeirão Temível, tributary of rioIpiranga, 24º7′36.00″S 47°59′29.00″W, Zawadzki, C. H. & Oliveira, C. A. M., 19 February 2009.

### 5.6 Characidium rachovii

Rio Uruguay basin: NUP 19088, 49, 11.4–36.9 mm *L*_S_, municipality of Alegrete, Arroio Carvonaraci, tributary of rio Ibicuí, 29°43′14.16″S 56°5′29.04″W, Deprá, G. C., Delvio, L. & Berriel, R., 12 February 2017. NUP 21952, 1, 25.0 mm *L*_S_, municipality of Itaqui, rio Ibicuí, 29°24′38.00″S 56º45′14.99″W, Massaro, M. V., 29 July 2019.

### 5.7 Characidium samurai

Rio das Almas basin: MZUSP 108188 (Holotype), 46.6 mm *L*_S_, municipality of Piraí do Norte, rio do Peixe, tributary of rio das Almas, 13°46′35″S 39°23′40″W, Oyakawa, O. T. *et al*., 13 August 2012.

### 5.8 Characidium satoi

Rio São Francisco basin: MZUSP 95289 (Paratype), 10, 36.3–41.8 mm *L*_S_, Córrego Curral das Éguas, at the border between mun. Três Marias and São Gonçalo do Abaeté, 18°07′13″S 45°24′52″W, Oyakawa, O. T. *et al*., 3 October 2007.

### 5.9 Characidium serrano

Rio Uruguay basin: NUP 20570, 1, 40.0 mm *L*_S_, municipality of Porto Vera Cruz, rio Uruguay, 27°42′01″S 54°53′47″W, Massaro, M. & Tataje, D., 15 November 2017.

### 5.10 Characidium schubarti

Upper rio Paraná basin: NUP 21021, 1, 43.7 mm *L*_S_, municipality of Jaguariaíva, rio das Samambaias, tributary to rio Jaguariaíva, 24°10′43.68″S 49°36′55.8″, Graça, W. J. *et al*., 17 July 2018. NUP 21072, 1, 60.9 mm *L*_S_, municipality of Jaguariaíva, rio das Lanças, tributary to rio Jaguariaíva, 24°10′43.68″S 49°36′55.8″W, Graça, W. J. *et al*., 19 July 2018.

### 5.11 Characidium summus

Rio Madeira basin: MZUSP 116954 (Holotype), 34.5 mm *L*_S_, municipality of Guajará‐Mirim, upper rio Pacaás Novos, tributary of rio Mamoré, 10°50′46.29″S 63°37′47.29″W, Hungria D. & Ribeiro, A. C., 17 December 2013.

### 5.12 Characidium tapuia

Rio Parnaíba basin: MZUSP 87516, 8, 12.95–26.94 mm *L*_S_, municipality of Balsas, Ribeirão Jenipapo, tributary of rio Balsas, 7°28′04″S 46°09′36″W, Akama A. & Baena E., 24 March 2005.

### 5.13 Characidium tenue

Rio Uruguay basin: NUP 19078, 3, 15.0–25.8 mm *L*_S_, municipality of Alegrete, Arroio Carvonaraci, tributary of rio Ibicuí, 29°24′38.00″S 56°45′14.99″W, Deprá, G. C.; Delvio, L.; Berriel, R., 12 February 2017.

### 5.14 Characidium travassosi

Rio Iguaçu basin: MZUSP 85940 (Holotype), 33.8 mm *L*_S_, municipality of Reserva do Iguaçu, rio das Torres, a small tributary of rio Jordão, 25°48′28″S, 51°59′01″W, Moreira, C. R., 10 January 2001. NUP 18877, 2, 49.4–53.7 mm *L*_S_, municipality of Inácio Martins, riacho Lageado, tributary to rio Iratim, 25°30′5″S, 51°10′10″W, Graça, W. J, 04 October 2015. NUP 19033, 1, 47.0 mm *L*_S_, municipality of Cascavel, rio Tormenta, tributary to rio Iguaçu, 25°06′09″S, 53°08′10″W, Delariva, R. L., 04 October 2015. NUP 24862, 1, 47.82 mm *L*_S_, municipality of Reserva do Iguaçu, rio Lageado das Torres, tributary to rio Jordão, 25°50′28.02″S, 51°50′29.96″W, Graça W. J. *et al*., 04 October 2015. Rio Ribeira de Iguape basin: MZUSP 68132 (Paratype), 12, 34.8–42.5 mm *L*_S_, municipality of Quatro Barras, rio Taquari, tributary of rio Capivari, 2 km ahead of Morada do Silêncio Chaminé da Serra da Ordem Rosa Cruz, 25°20′20″S, 48°55′46″W, Oyakawa O. T. *et al*., 10 March 2001.

### 5.15 Characidium xanthopterum

Upper rio Paraná basin: NUP 4414 (Paratype), 15, 32.8–40.4 mm *L*_S_, municipality of Corumbaíba, córrego Gameleira, tributary to left margin of rio Corumbá, 17°59′S 48°29′W, Nupélia staff, 12 October 1996.

## AUTHOR CONTRIBUTIONS

Conceptualization: B.H.M.S., R.B.R., and W.J.G. Developing methods: B.H.M.S. and R.B.R. Conducting the research, data analysis, and data interpretation: all authors. Writing: all authors. Funding: B.H.M.S., R.B.R., W.J.G., and A.V.O.

## FUNDING INFORMATION

W.J.G. is supported by CNPq (Conselho Nacional de Desenvolvimento Científico e Tecnológico, MCTI‐Brazil) with productivity scholarships (305200/2018‐6 and 307089/2021‐5). R.B.R. and B.H.M.S. are supported by a scholarship from Coordenação de Aperfeiçoamento de Pessoal de Nível Superior‐Brasil (CAPES) process numbers 88887.629034/2021‐00 and 88887.629037/2021‐00, respectively.

## Supporting information


**TABLE S1.** Molecular data and taxonomic identification of *Characidium* species used in this study.

**TABLE S2.** Inter‐ and intraspecific K2P mean distance within groups of *Characidium* based on cytochrome *c* oxidase subunit I (*COI*) sequences.
